# Duffy Antigen Receptor for Chemokines Mediates Chemokine Endocytosis through a Macropinocytosis-Like Process in Endothelial Cells

**DOI:** 10.1371/journal.pone.0029624

**Published:** 2011-12-27

**Authors:** Yani Zhao, Nilam S. Mangalmurti, Zeyu Xiong, Bharat Prakash, Fengli Guo, Donna B. Stolz, Janet S. Lee

**Affiliations:** 1 Division of Pulmonary, Allergy, and Critical Care Medicine, Department of Medicine, University of Pittsburgh, Pittsburgh, Pennsylvania, United States of America; 2 Pulmonary, Allergy and Critical Care Division, Department of Medicine, University of Pennsylvania, Philadelphia, Pennsylvania, United States of America; 3 Department of Cell Biology and Physiology, Center for Biologic Imaging, University of Pittsburgh, Pittsburgh, Pennsylvania, United States of America; Ottawa Hospital Research Institute, Canada

## Abstract

**Background:**

The Duffy antigen receptor for chemokines (DARC) shows high affinity binding to multiple inflammatory CC and CXC chemokines and is expressed by erythrocytes and endothelial cells. Recent evidence suggests that endothelial DARC facilitates chemokine transcytosis to promote neutrophil recruitment. However, the mechanism of chemokine endocytosis by DARC remains unclear.

**Methodology/Principal Findings:**

We investigated the role of several endocytic pathways in DARC-mediated ligand internalization. Here we report that, although DARC co-localizes with caveolin-1 in endothelial cells, caveolin-1 is dispensable for DARC-mediated ^125^I-CXCL1 endocytosis as knockdown of caveolin-1 failed to inhibit ligand internalization. ^125^I-CXCL1 endocytosis by DARC was also independent of clathrin and flotillin-1 but required cholesterol and was, in part, inhibited by silencing Dynamin II expression**.** ^125^I-CXCL1 endocytosis was inhibited by amiloride, cytochalasin D, and the PKC inhibitor Gö6976 whereas Platelet Derived Growth Factor (PDGF) enhanced ligand internalization through DARC. The majority of DARC-ligand interactions occurred on the endothelial surface, with DARC identified along plasma membrane extensions with the appearance of ruffles, supporting the concept that DARC provides a high affinity scaffolding function for surface retention of chemokines on endothelial cells.

**Conclusions/Significance:**

These results show DARC-mediated chemokine endocytosis occurs through a macropinocytosis-like process in endothelial cells and caveolin-1 is dispensable for CXCL1 internalization.

## Introduction

The malarial parasite receptor and minor blood group antigen, Duffy, is a chemokine binding protein expressed on erythrocytes and the surface of post-capillary venular endothelial cells [1], [2], [3]. Unlike other heptahelical receptors, Duffy Antigen Receptor for Chemokines (DARC), lacks a G-coupling protein motif and therefore does not participate in G-protein mediated signaling [4]. As ligation of erythrocyte Duffy by chemokines renders chemokines inaccessible to circulating neutrophils, the concept of DARC as a chemokine sink was established [1]. However, the role of DARC appears to be more expansive, as we and others have established that both erythrocyte and endothelial DARC can modulate the inflammatory response and chemokine-mediated neutrophil recruitment during inflammatory states [1], [5], [6], [7], [8], [9].

The findings of enhanced expression of DARC on post capillary venular endothelium and capillaries of the lungs during inflammatory states further support DARC's role in inflammation [10]. We have previously shown that endothelial DARC is up-regulated in the capillaries of human lungs during suppurative pneumonia, a condition characterized by intense neutrophilic inflammation [10]. Furthermore, we have also shown that DARC facilitates the movement of radiolabeled ^125^I -CXCL1/GRO-α across an endothelial monolayer and augments neutrophil recruitment to inflammatory sites [5]. Consistent with this finding, others have shown that Duffy antigen mediates chemokine endocytosis [8]. Interestingly, once internalized by DARC, chemokine was not degraded but transcytosed and retained on the apical surface of the endothelium, where it can be presented to circulating neutrophils and thus participate in neutrophil recruitment during inflammatory states [8].

In endothelial cells, DARC has been detected within membrane invaginations that have the appearance of caveolae [11]. CXCL8, a known Duffy ligand, has been shown to localize within caveolar vesicles following endocytosis [12]. Recent studies have also demonstrated that DARC mediates chemokine transcytosis across the endothelium and co-localizes with CCL2 and caveolin-1 in vesicles [8]. However, direct biochemical and functional evidence of a caveolin-dependent pathway utilized by DARC are lacking and the exact mechanisms of DARC mediated chemokine internalization are unknown. We therefore sought to determine the mechanisms of DARC-mediated chemokine transcytosis.

## Results

**Figure 1:**
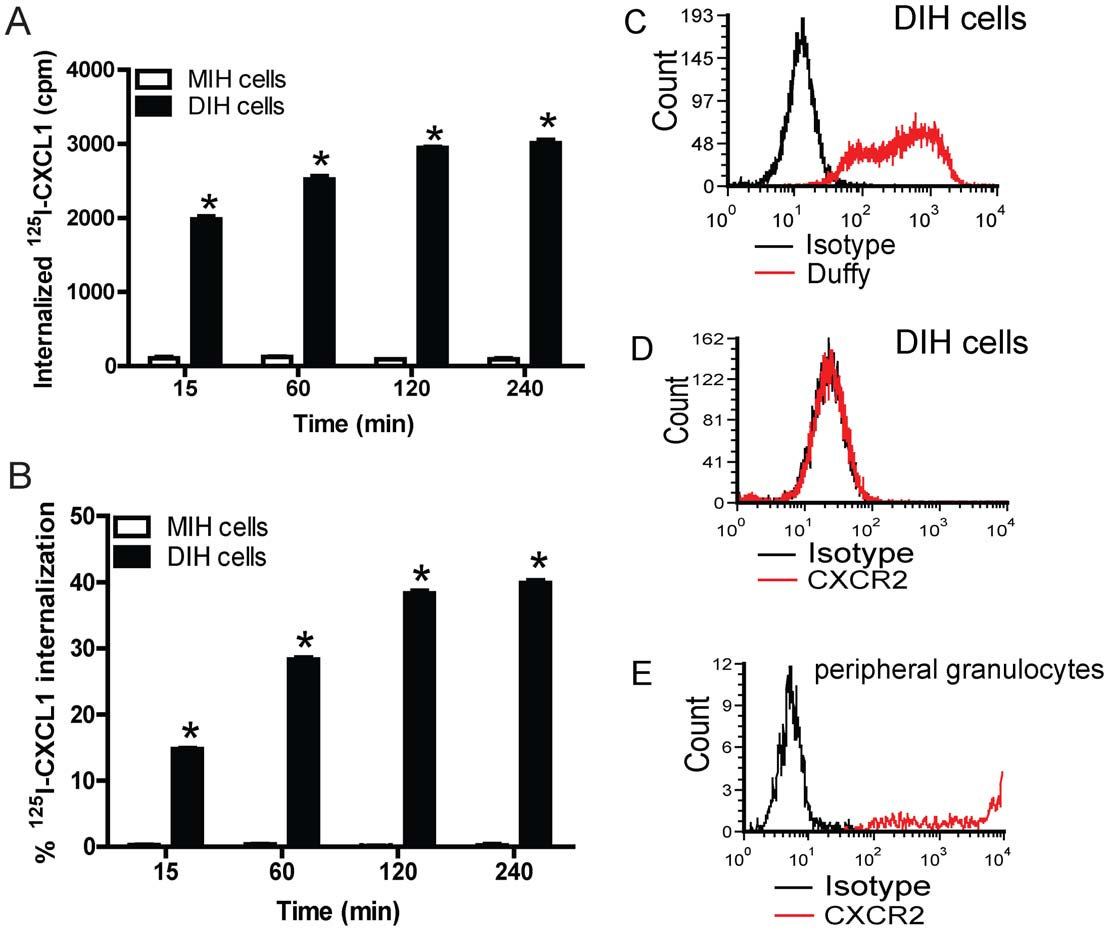
Duffy antigen mediates CXCL1 binding and endocytosis in DIH cells. MIH cells and DIH cells were incubated with ^125^I-CXCL1/GRO-α at the designated time points then were washed with binding buffer or acid wash buffer, as described in Materials and Methods. (A) Internalized ^125^I-CXCL1/GRO-α counts in MIH and DIH cells. * *p*<0.01 MIH cells *vs* DIH cells, paired t test. (B) Percentage of ^125^I-CXCL1/GRO-α internalization in MIH and DIH cells. * *p*<0.001 MIH cells *vs* DIH cells, paired t test. Experiments were conducted in triplicates and the mean ± SEM from two independent experiments is shown. (C) DIH cells were labeled with anti-Duffy 2C3 Fy6 antibody or isotype mouse IgG1 antibody and surface expression of Duffy examined by flow cytometry. (D) DIH cells do not show detectable surface CXCR2 expression following immune-labeling with anti-human CXCR2 or isotype mouse IgG1 antibody. (E) Peripheral leukocytes were isolated from a healthy volunteer and immune-labeled with anti-human CXCR2 or isotype IgG1 control antibody. Histogram shows gated population of granulocytes expressing CXCR2. Flow cytometric examination of surface DARC and CXCR2 expression on DIH cells was performed 2 independent times. CXCR2 expression on human peripheral granulocytes was performed once.

As expression of DARC in cultured endothelial cells is rapidly lost, we stably expressed DARC cDNA into an immortalized human umbilical vein endothelial cell (HUVEC) line [5], [12], [13]. We previously reported that the Duffy-expressing immortalized HUVEC (DIH) shows saturable binding of ^125^I-CXCL1/GRO-α with equilibrium dissociation constant (Kd) of ∼5 nM and binds multiple chemokines with a binding profile consistent with what is reported *in vivo* [3], [14], [15], [16], [17], [18]. Here, we studied the rate of chemokine-ligand internalization in DIH and mock-transfected immortalized HUVEC (MIH) cells. We took advantage of the fact that receptor-ligand interactions on the cell surface are disrupted at low pH and that internalization is a temperature sensitive process [15], [19], [20]. MIH cells did not bind or internalize ^125^I-CXCL1/GRO-α whereas DIH cells showed rapid ^125^I-CXCL1 internalization by 15 minutes with further increases up to 240 minutes (Fig. 1A). However, most of the ligand could be removed by acid stripping and approximately 40% of bound ligand internalized by 240 minutes (Figure 1B). To determine whether the modest % of ligand endocytosis was related to reduced cell integrity over time, we measured cell viability by trypan blue exclusion and by flow cytometric analysis of 7-aminoactinomycin D (7-AAD)+ cells. Greater than 99% of the cells remained viable at 240 minutes by trypan blue exclusion, and 95% of the cells remained 7AAD negative at 240 minutes by flow cytometric analysis (data not shown). In addition, the internalization rate in DIH cells was similar to Human Erythroleukemia cells (HEL) which natively express Duffy antigen (about 30% ligand internalization by 240 minutes) (data not shown).

Because CXCR2 is a high affinity receptor for CXCL1 previously reported to be expressed on endothelial cells [21] and can potentially influence DARC-dependent CXCL1 endocytosis, we examined for surface CXCR2 expression on DIH cells by flow cytometry (Figure 1C-E). Although DIH cells express surface DARC (Figure 1C), we were unable to confirm detectable surface CXCR2 expression (Figure 1D). This is in contrast to peripheral granulocytes which expressed varying levels of surface CXCR2 under basal conditions (Figure 1E). Collectively, these data show that ^125^I-CXCL1 binding and internalization is dependent upon DARC in DIH cells.

### Depletion of cellular cholesterol inhibits DARC-mediated CXCL1 internalization in endothelial cells

**Figure 2:**
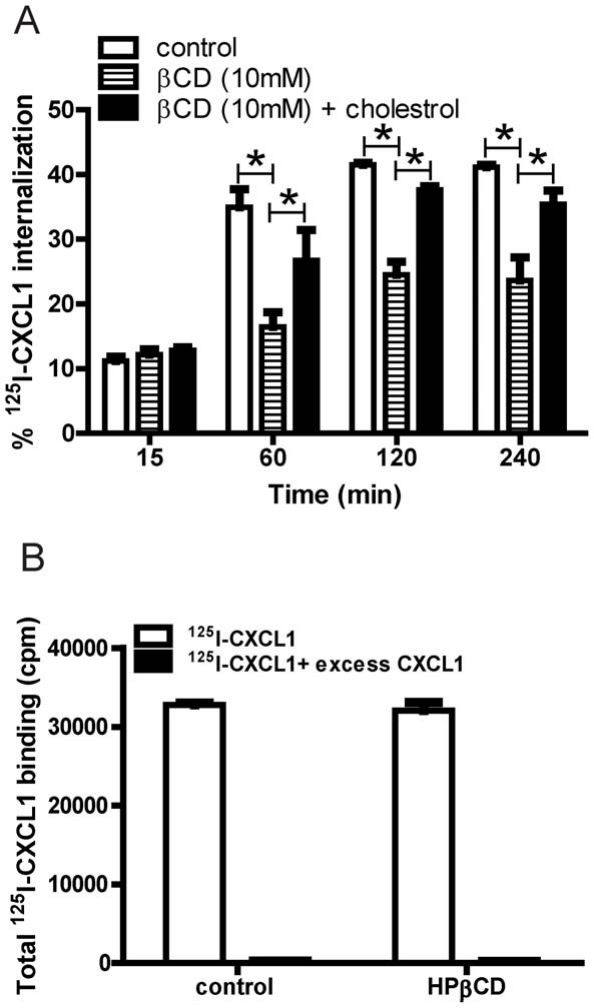
Cholesterol is required for CXCL1 endocytosis in DIH cells. (A) DIH cells were pre-treated with 2-hydroxypropyl-β-Cyclodextrin prior to incubation with ^125^I -CXCL1/GRO-α for various time points, as described in the Materials and Methods. 2-hydroxypropyl-β-cyclodextrin showed inhibition of chemokine internalization that could be reversed with cholesterol repletion. * *p*<0.01, 10 mM 2-hydroxypropyl-β-Cyclodextrin treatment *vs* control, two way ANOVA. ** *p*<0.05 10 mM 2-hydroxypropyl-β-Cyclodextrin treatment *vs* repletion with cholesterol, two way ANOVA. (B) DIH cells were pre-treated with 2-hydroxypropyl-β-Cyclodextrin, prior to the addition of ^125^I -CXCL1/GRO-α with or without unlabeled excess CXCL1 for 120 minutes, as described in the Materials and Methods. Data presented are the combined results from at least three independent experiments.

Cholesterol is involved in a number of endocytic pathways, including the caveolin-dependent pathway, invagination of clathrin coated pits, membrane ruffling and macropinocytosis [22], [23], [24], [25], [26], [27]. We therefore used 2-hydroxypropyl-β-cyclodextrin (HPβCD) to deplete cholesterol from the plasma membrane in order to determine whether the mechanism of ligand internalization through DARC was dependent upon cholesterol. HPβCD treatment significantly attenuated CXCL1 internalization by DIH cells (Figure 2A). The effect of HPβCD was not due to altered binding of CXCL1 by DIH cells, as we could displace similar amounts of surface bound ^125^I-CXCL1 by excess unlabeled CXCL1 in both HPβCD treated and untreated cells (Figure 2B). Cholesterol repletion reduced the inhibitory effect of HPβCD pre-treatment on chemokine endocytosis, with near complete restoration of chemokine endocytosis to basal conditions by 4 h (p<0.05) (Figure 2A). These data demonstrate that DARC-mediated chemokine internalization is dependent upon cholesterol.

### Caveolin-1 is not essential for DARC-mediated ligand internalization in endothelial cells

**Figure 3:**
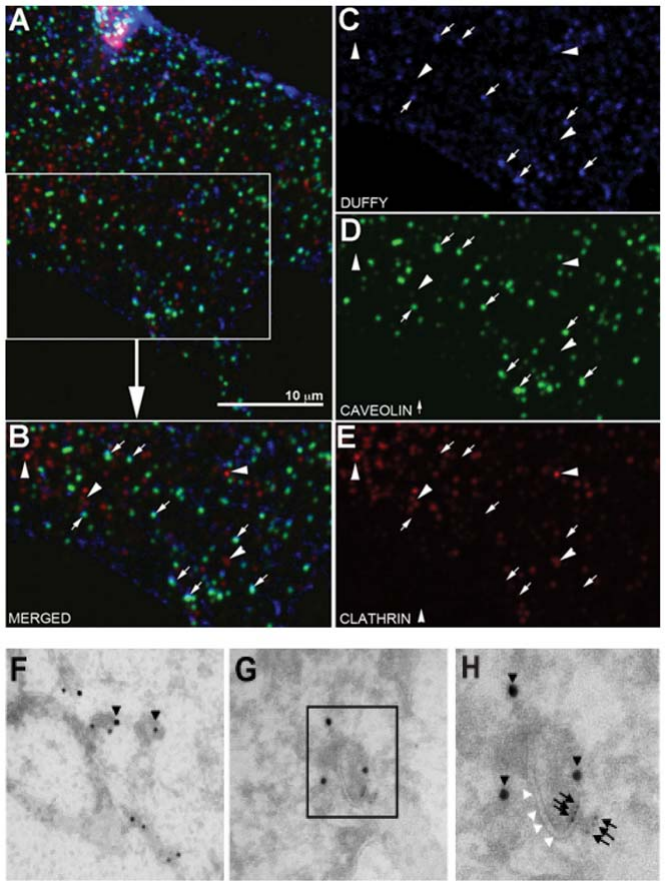
Duffy antigen co-localizes with caveolin-1 but not with clathrin. The DIH cells were stimulated with or without CCL2 100 ng/ml for 120 minutes. Figure A shows a typical single plane confocal optical section from the lamellipodial region of a cell, with Panel B focusing at a specific area on the cell outlined by the rectangle in A. Panel B is then separated into specific color signals to accentuate the localization status of each protein. Cells are stained for Duffy (Panel C, blue), caveolin (Panel D, green) and clathrin (Panel E, red). Some Duffy positive foci colocalize with the caveolin signal (arrows) while no Duffy signal is seen colocalized with the red clathrin signal(arrowheads). Figure (F-H), by immuno-EM, caveolin-1 was labeled with 10 nm gold particle- (▾), Duffy antigen was labeled with 5 nm gold particle- (**↓**), colocalization between caveolin and Duffy is observed in flask-like membrane structures- (Δ).

As caveolae-mediated endocytosis is disrupted by cholesterol depletion and caveolin-1 has been shown to co-localize with the DARC ligands CXCL8 and CCL2 in addition to DARC, we investigated whether DARC-mediated ligand endocytosis occurs through a caveolae-dependent pathway [8], [12]. We examined intracellular localization of DARC, caveolin-1, and clathrin in DIH cells in the presence CCL2 (Figure 3A-E). Consistent with the findings of Pruenster and colleagues, we show that DARC co-localized with caveolin-1, a marker of caveolae, but not clathrin by confocal microscopy in DIH cells (Figure 3A-E) [8]. Similar findings were obtained when CXCL1 was used as the ligand (data not shown). By immunoelectron microscopy, we confirm that caveolin-1 (labeled with 10 nm gold particles, black triangle) co-localized with DARC (labeled with 5nm gold particle, black arrows) in DIH cells (Figure 3F, G and H).

**Figure 4:**
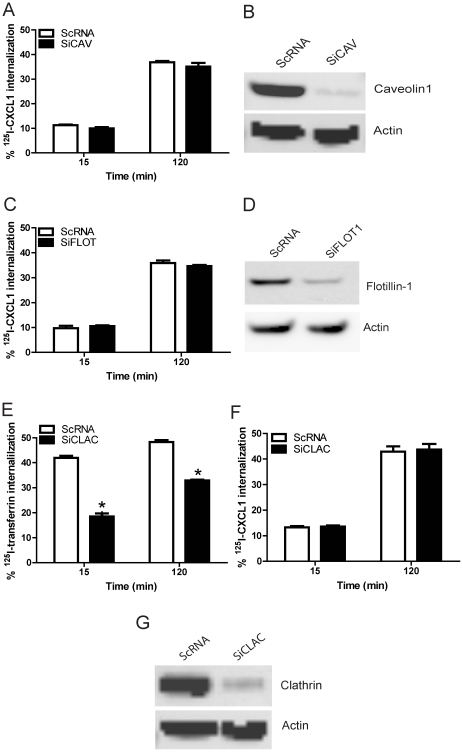
Duffy mediated chemokine endocytosis in DIH cells is caveolin-1, clathrin, flotillin-1 independent. DIH cells were transfected with SiRNA targeted against caveolin-1 (A&B), flotillin-1(C&D), clathrin (E, F &G), and control scramble sequence RNA (ScRNA). At 72 hour post-transfection, endocytosis experiments were performed and cells lysates were analysed by SDS-PAGES and immunoblotted with caveolin-1, clathrin, flotillin-1 and β-actin antisera. (E) DIH cells transfected with SiCLAC or control SiRNA and were incubated with ^125^I-transferrin for various time points then were washed with binding buffer or acid wash buffer, as described in the Materials and Methods. The mean ± SEM from three independent experiments is shown.

To determine whether caveolae are required for DARC-mediated chemokine internalization, we transfected DIH cells with siRNA targeted against caveolin-1 and control siRNA. Following transfection, immunoblotting was performed to confirm knockdown of caveolin-1. Near complete knockdown of caveolin-1 expression had no effect on ^125^I-CXCL1 internalization in DIH cells (Figure 4A–B). We next examined whether flotillin-1, a protein implicated in cholesterol-dependent, lipid raft-associated pathways [28], participates in the endocytosis of chemokine in Duffy expressing endothelial cells. The depletion of flotillin-1 had no effect on ^125^I-CXCL1 internalization compared to the cells transfected with control siRNA (Figure 4C–D). In contrast, knockdown of clathrin significantly attenuated ^125^I-transferrin uptake (Figure 4E) but had no effect on chemokine endocytosis in our model (Figure 4F), confirming that DARC-mediated chemokine endocytosis does not utilize a clathrin-dependent pathway. Notably, the majority of transferrin uptake was rapid occurring by 15 minutes whereas CXCL1 endocytosis occurred more slowly. The % rate of internalization in our model, however, for transferrin and CXCL1 was comparable by 120 minutes. Collectively, these findings show that although DARC co-localizes with caveolin-1, an essential component of caveolae, caveolin-1 in addition to flotillin-1 and clathrin are dispensable for DARC-mediated chemokine endocytosis. However, ligand internalization is dependent upon cholesterol, suggesting that alternative cholesterol-mediated processes such as the macropinocytic pathway may participate in DARC-mediated ligand internalization in endothelial cells [23], [29].

### DARC is located along plasma membrane extensions and PDGF enhances DARC-mediated chemokine endocytosis

**Figure 5:**
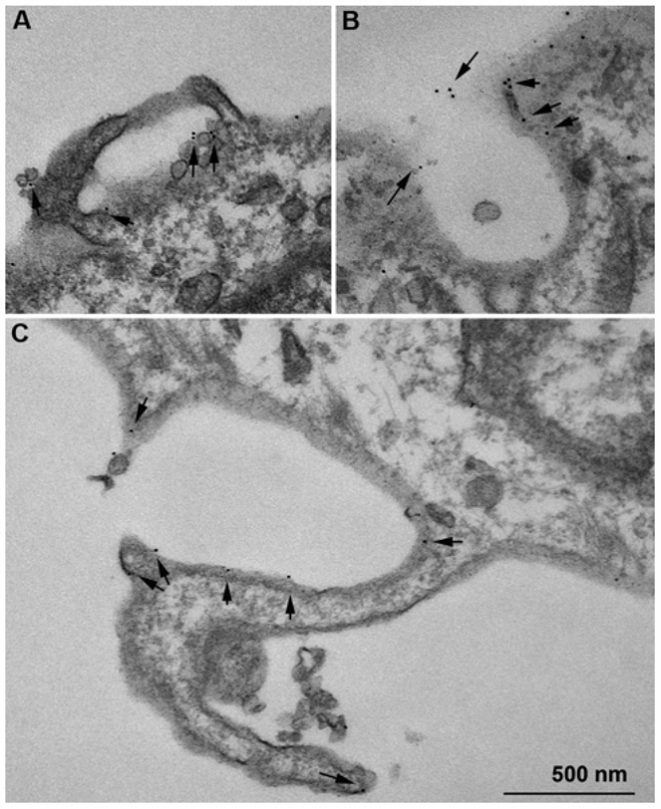
Majority of Duffy antigen is present on the surface of endothelial cells within vesicles and plasma membrane extensions. Duffy antigen was detected on membrane surfaces by pre-embedding TEM in DIH cells as described in Materials and Methods. All panels are imaged from different cells. Duffy antigen (5 nm gold, arrows) was seen located associated with plasma membrane extensions and invaginations. These structures are large (>100 nm) and have no consistent morphology, suggesting they are macropinosomes.

**Figure 6:**
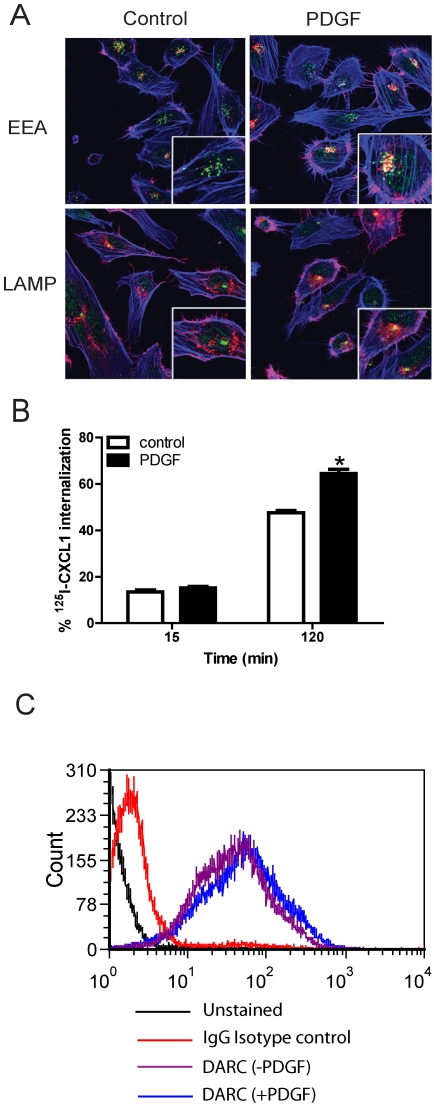
PDGF enhances chemokine internalization in DIH cells and co-localization with early endosomal antigen. (A) DIH cells were stimulated with or without PDGF for 10 min, then DARC (red) and EEA (green) or LAMP (green) co-localization were detected using confocal imaging. Blue, actin. (B) DIH cells were pretreated with PDGF 50 ng/ml for 10 min prior to endocytosis experiments. By 120 minutes, PDGF increased DARC-mediated CXCL1 internalization. * *p*<0.05 PDGF treatment *vs* control, paired t test. Cells incubated in the absence of PDGF served as a control for internalization. Data presented are the combined results of at least three independent experiments. (C) DIH cells were pretreated with or without PDGF 50 ng/ml for 10 min prior to surface staining for DARC as described in the Materials and Methods.

Actin-mediated plasma membrane ruffling is a morphological change occuring early in several endocytic pathways, including circular dorsal ruffles (CDRs) that lead to macropinosome formation [30]. We therefore evaluated the subcelluar localization of DARC using transmission electron microscopy in resting DIH cells. DARC was localized to the surface of endothelial cells, located along plasma membrane extensions with the appearance of ruffles (Fig. 5). In response to growth factor stimulation, ruffling occurs within minutes and mediates receptor tyrosine kinase internalization via the CDRs endocytic pathway [31]. As PDGF treatment is a known inducer of ruffling formation [32], and our findings demonstrate the presence of DARC in membrane extensions with the appearance of ruffles, we investigated the effect of PDGF on chemokine internalization in DIH cells. Confocal imaging confirmed DARC internalization as DARC was associated with early endosomal antigen (EEA), and to a lesser extent with lysosomal associated membrane protein (LAMP) following PDGF stimulation (Figure 6A). PDGF stimulation consistently increased ^125^I-CXCL1 internalization in endothelial cells by 120 minutes (Figure 6B). The effect of PDGF is not due to upregulation of DARC expression or increased mobilization of DARC to the surface of the DIH cells (Figure 6C). These data show that a growth factor mediated pathway enhances CXCL1 internalization in DIH cells.

### DARC-mediated CXCL1 internalization does not require PAK1 and CtBP1 but is dependent upon Dynamin II expression

**Figure 7:**
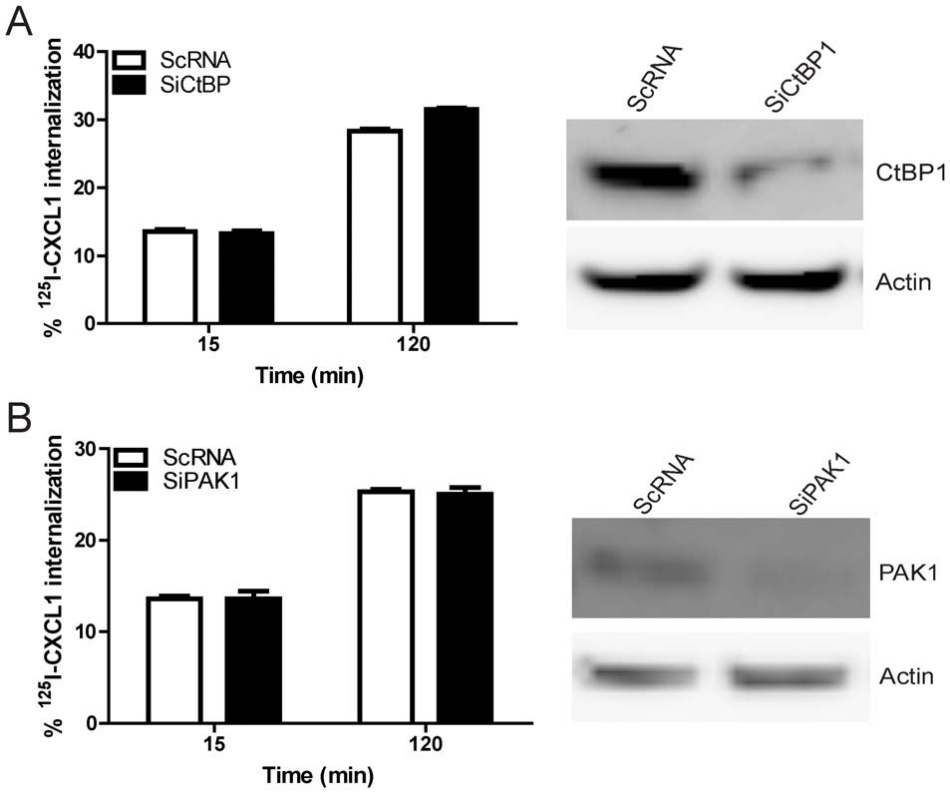
CXCL1 endocytosis is CtBP1 and PAK1 independent. DIH cells were transfected with SiRNA targeted against CtBP1 (A) or PAK1 (B) and control scramble sequence RNA (ScRNA). At 72 hour post-transfection, endocytosis experiment was performed and cells lysates were analysed by SDS-PAGES and immunoblotted with CtBP1, PAK1 and β-actin antisera. The mean ± SEM from three independent experiments is shown.

It has been shown that P21-activated kinase 1 (PAK1) is associated with membrane ruffling and it is thought to play an important regulatory role in macropinocytosis [33]. Phosphorylation of C-terminal-binding protein-1 (CtBP1) occurs by activated PAK1 and CtBP1 is essential for the final fission of PAK1 dependent macropinosomes from the plasma membrane [34]. We therefore examined the role of PAK1 and CtBP1 in CXCL1 internalization by DIH cells. As compared with HeLa cells known to express abundant PAK1, DIH cells showed minimal PAK1 expression (data not shown). In contrast, CtBP1 expression in DIH cells was easily detectable under basal conditions (Figure 7A). Silencing of either PAK1 or CtBP1 did not effect chemokine internalization by DIH cells (Fig. 7A&B). Based upon these findings, we concluded that DARC-ligand internalization in DIH cells is PAK1 and CtBP1 independent.

**Figure 8:**
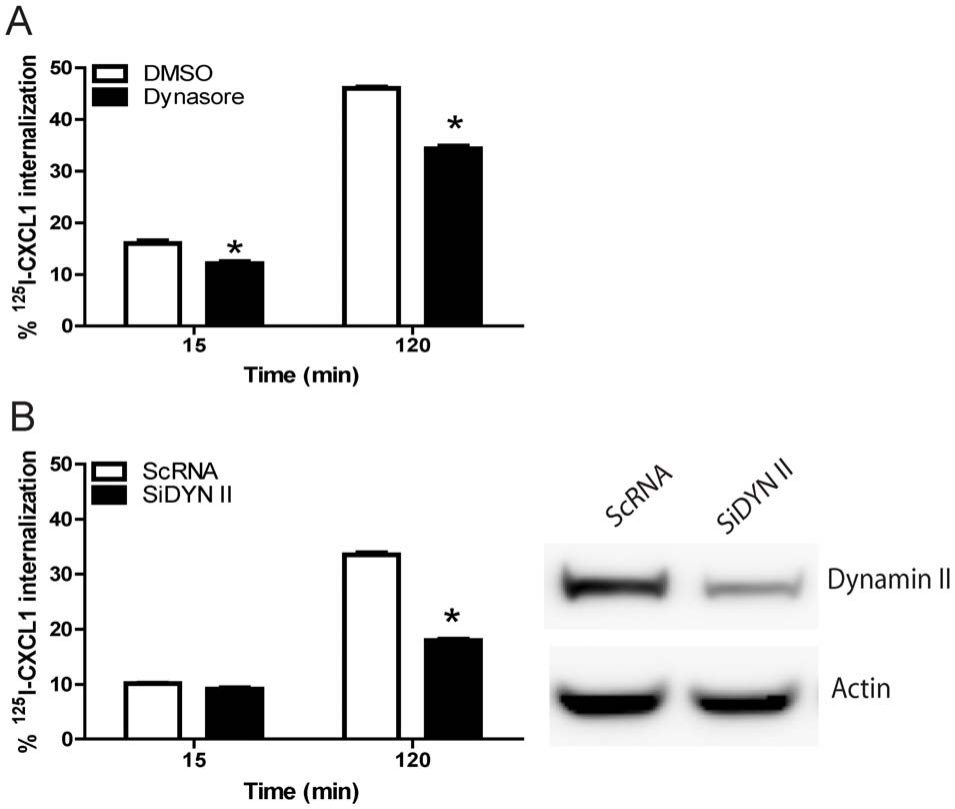
Silencing of Dynamin II expression partially inhibited chemokine endocytosis. (A) DIH cells were pretreated with dynasore prior to incubation with ^125^I-CXCL1/GRO-α for the specified time points, as described in Materials and Methods. Dynasore inhibited CXCL1 internalization in DIH cells. * *p*<0.05 dynasore treatment *vs* control, paired t test. (B) DIH cells were transfected with SiRNA targeted against dynamin II and control SiRNA (ScRNA). At 72 hour post-transfection, endocytosis experiments were performed and cells lysates were analysed by SDS-PAGES and immunoblotted with dynamin II and β-actin antisera. The mean ± SEM from three independent experiments is shown.

Dynamin II is a GTPase that promotes fission of the endocytic membrane and is required for both clathrin and caveolae mediated endocytosis [35], [36]. Dynamin II may also be involved in regulating actin polymerization and ruffle formation during macropinocytosis [37]. To assess if dynamin is involved in CXCL1 endocytosis in DIH cells, DIH cells were treated with dynasore, an inhibitor of dynamin. Dynasore treatment showed inhibition of internalization of CXCL1 (Figure 8A). Confirming these results, knockdown of Dynamin II expression by siRNA also inhibited CXCL1 endocytosis by 120 minutes (Figure 8B). These data suggest that dynamin II participates in DARC mediated CXCL1 endocytosis.

### CXCL1 endocytosis in DIH cells requires actin polymerization, is amiloride sensitive, and PKC dependent

**Figure 9:**
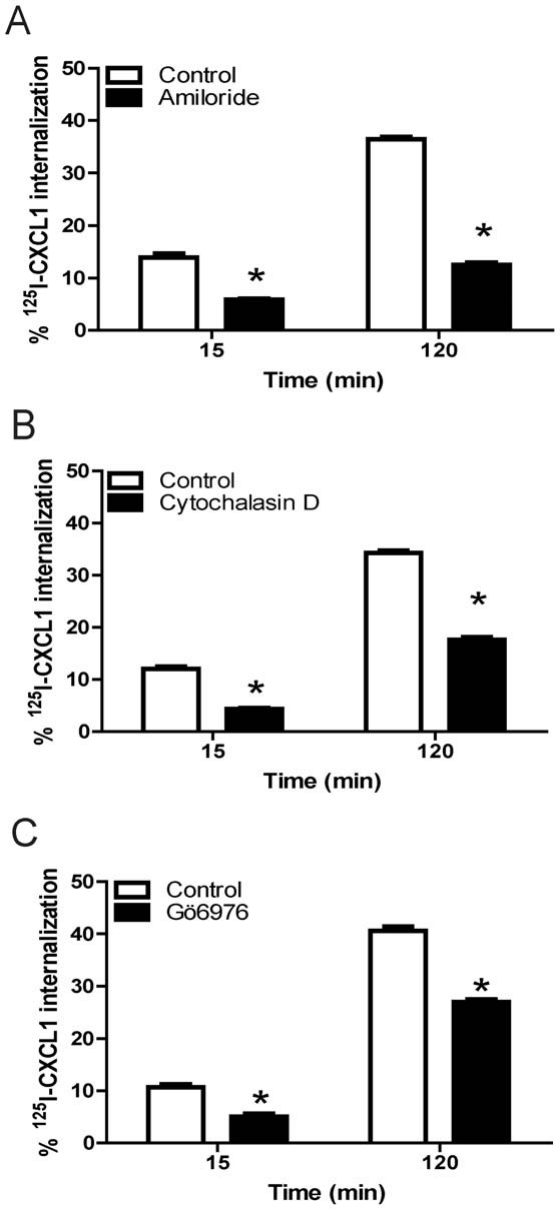
Addition of amiloride, inhibition of actin polymerization, or PKC inhibition decreased CXCL1 internalization in DIH cells. DIH cells were pre-treated with various inhibitors prior to incubation with ^125^I-CXCL1/GRO-α for the specified time points, as described in Materials and Methods. (A) Amiloride significantly inhibited DARC-mediated CXCL1 internalization in DIH cells. * *p*<0.05 amiloride treatment *vs* control, paired t test. (B) Actin polymerization inhibitor cytochalasin D decreased CXCL1 internalization in DIH cells. * *p*<0.05 cytochalasin D treatment *vs* control, paired t test. (C) PKC inhibitor Gö6976 also showed inhibition of CXCL1 internalization. * *p*<0.05 Gö6976 treatment *vs* control, paired t test. Shown is the mean ± SEM from three experiments.

Our findings suggest that DARC-mediated CXCL1 endocytosis is dependent on both cholesterol and Dynamin II and is stimulated by the growth factor, PDGF. As these molecules have been previously implicated in macropinocytosis, we examined whether CXCL1 internalization in DIH cells occurred through this pathway. Amiloride, an ion exchange inhibitor, is widely used as an inhibitor of macropinocytosis, as it inhibits macropinocytosis without a detectable impact on the other known endocytic pathways [24], [38]. Amiloride significantly reduced CXCL1 internalization in DIH cells (Fig. 9A), suggesting that endothelial CXCL1 internalization occurs through macropinocytosis-like process.

Membrane ruffling, an essential feature of macropinocytosis, occurs following actin polymerization [24]. We therefore investigated the effects of actin disruption on CXCL1 internalization using cytochalasin D, a known inhibitor of actin polymerization [39]. Cytochalasin D treatment significantly attenuated CXCL1 internalization in DIH cells (Fig. 9B). Because protein kinase C (PKC) has been shown to be a regulator of macropinocytosis [40], [41], we tested the effect of PKC inhibition on chemokine internalization in DIH cells. As shown in Fig. 9C, the selective PKC inhibitor Gö6976 inhibited CXCL1 internalization compared to untreated cells. In addition, wortmannin, a PI3K inhibitor, also inhibited ligand endocytosis by 15 minutes (p<0.05, data not shown). Taken together, these data suggest a macropinocytosis-like process is involved in DARC-induced CXCL1 internalization in endothelial cells and actin and PKC mediate this process.

## Discussion

The mechanism of DARC-mediated chemokine endocytosis in endothelial cells remains poorly defined. In this study, we show several novel findings exploiting the high affinity ligand-receptor interaction between DARC and the pro-angiogenic CXCL1. Despite previous reports and our finding showing DARC localized to caveolae, caveolin-1 is dispensable for DARC-mediated chemokine endocytosis. In addition, clathrin does not appear essential for DARC-dependent ligand internalization. Flotillin-1, an endosomal protein involved in clathrin-independent and caveolae-independent endocytosis, is also not essential [28]. Our findings further show that the majority of DARC-chemokine interactions occur along the surface of DIH cells, located along plasma membrane extensions, with ligand internalization enhanced by PDGF stimulation. The data implicate an alternative cholesterol-dependent pathway for DARC-ligand internalization that requires, in part, dynamin II. Moreover, chemokine endocytosis also requires actin polymerization, is amiloride sensitive, and PKC-dependent. Although PAK1- and CtBP1-independent, these lines of evidence support a macropinocytosis-like process mediating ligand endocytosis by DARC.

There are multiple endocytic pathways employed by endothelial cells including clathrin-dependent and clathrin-independent mechanisms. Clathrin-mediated endocytosis has been the most extensively studied mechanism of chemokine internalization [42], [43], [44]. CXCR1 and CXCR2 mediate chemokine endocytosis through a clathrin, Rab5, dynamin II-dependent process [45]. A clathrin-dependent pathway is utilized in the internalization of D6, a seven transmembrane non-signaling chemokine receptor similar to DARC [46]. Lipid raft and caveolae-mediated pathways of chemokine internalization have also been described for the chemokine receptors CCR5 and CXCR4 [42], [47], [48]. Other clathrin-independent pathways less commonly utilized by endothelial cells include flotillin-mediated endocytosis, phagocytosis, clathrin independent cargos/GPI-AP-enriched early endosomal compartment (CLIC/GEEC) endocytic pathway, and macropinocytosis [28], [49], [50], [51]. Our findings are the first report of DARC, a seven transmembrane chemokine binding protein, preferentially utilizing a macropinocytosis-like process for ligand internalization.

A number of endocytotic pathways require lipid rafts and can be inhibited by the cholesterol-depleting agent cyclodextrin. We found that treatment of DIH cells with HPβCD inhibited CXCL1 internalization. Cholesterol repletion following HPβCD treatment restored chemokine endocytosis, demonstrating that cholesterol is required for Duffy antigen mediated CXCL1 internalization. Caveolae-dependent endocytosis requires intact lipid rafts and is inhibited by depletion of cholesterol. Duffy antigen has previously been detected in caveolae and colocalizes with the chemokine CCL2 in caveolin containing vesicles [8], [11]. In this study, however, gene silencing of caveolin-1 expression had no effect on DARC-mediated chemokine internalization. Although the findings demonstrate that caveolae is not the dominant mode of DARC-mediated CXCL1 entry, it remains possible that constitutive re-cycling of DARC in the absence of ligand stimulation may utilize caveolae. Our findings also do not exclude the possibility that DARC utilizes multiple redundant pathways for ligand internalization, and that knockdown of one pathway re-routes DARC to another pathway.

It is well established that dynamin plays an important role in both clathrin and caveolae mediated endocytosis [35], [36], [50]. However, dynamin is also involved in macropinocytosis as it is localized to pinosome-associated actin comet tails in several types of cells [52], [53]. Here, we demonstrate that inhibitors of dynamin II and genetic knockdown of dynamin II consistently reduced chemokine internalization by approximately 50% by 120 minutes, suggesting that Duffy mediated chemokine internalization is at least partially dependent on dynamin. Because we were unable to achieve complete knockdown of dynamin II despite higher molar concentrations of siRNA used (∼ 80 nM), we cannot rule out the possibility that even low expression of dynamin II in endothelial cells is sufficient to mediate DARC-ligand internalization.

In our model, CXCL1 internalization was inhibited by amiloride and enhanced by PDGF stimulation, two defining features of macropinocytosis. Furthermore, inhibition of actin polymerization and PKC activity consistently reduced chemokine internalization, supporting the concept that a macropinocytosis-like process is involved in DARC-mediated chemokine internalization. CtBP1 and PAK1 are involved in the final fission of the macropinosome from the plasma membrane in macropinocytosis [24], [34]. However, depletion of CtBP1 and PAK1 expression by siRNA did not attenuate ligand endocytosis in DIH cells. One explanation for the differing results may be that our studies were conducted in a different cell type from those in previous reports. The macropinocytosis process is still incompletely characterized, and unlike receptor-mediated endocytosis and phagocytosis, is not currently defined by a specific molecular pathway [24]. It is unclear how many system variations of macropinocytosis there are in different cell types, as theoretically almost all cell types are capable of macropinocytosis [54]. Moreover, there are different kinds of ruffles such as the canonical planar ruffle and circular ruffles. However, circular ruffles do not appear to require the Rho family GTPase Ras and Rac1 which are upstream of the target protein PAK1 [24], and this may account for why we were unable to block ligand endocytosis by targeting PAK1 or CtBP1.

In summary, our findings provide functional evidence that DARC-mediated ligand endocytosis is dependent upon a macropinocytosis-like process in endothelial cells and that caveolin-1 is dispensable for CXCL1 internalization. We also show that the majority of DARC-ligand interactions occur on the endothelial surface, supporting the concept that DARC provides a high affinity scaffold for surface retention of chemokines on endothelial cells. This is consistent with the findings reported by others that DARC internalizes and facilitates the transfer of ligand leading to their apical retention [8]. Recently, DARC has been shown to exist as homo-dimers and antagonize CCR5 signaling through the formation of hetero-oligomers [55]. It remains to be seen whether the process of hetero-oligomer formation diverts DARC to different endocytic pathways than dimerization.

## Materials and Methods

### Cell Culture

We previously generated and functionally characterized immortalized Human Umbilical Vein Endothelial Cells (HUVEC) stably expressing human Duffy cDNA (Accession no. AY167991) [5]. The immortalized HUVEC line (IVEC) has an indefinite lifespan in culture but is nontumorgenic. The cell line demonstrates cobblestone morphology, expresses Factor VIII-related Antigen, takes up Dil-Ac-LDL, and expresses the integrin subunits αvβ3, αvβ5, α2, α3, β4, and α6, consistent with its endothelial origin. The Duffy transfected immortalized HUVEC cells are herein referred to as DIH (Duffy transfected Immortalized HUVEC) cells. The mock transfected immortalized HUVEC cells are referred to as MIH (Mock transfected Immortalized HUVEC) cells. Endothelial cells were maintained in endothelial growth medium-2 (EGM2) containing 2% fetal calf serum (BioWhittaker, Walkersville, MD). Upon passage, these cells were repeatedly tested negative for mycoplasma by PCR.

### Immunofluorescence Confocal microscopy

DIH cells were grown in 24 well plates containing 12 mm round glass coverslips (Thermo-Fisher, Pittsburgh, PA). Cells were stimulated with 100 ng/mL human recombinant CCL2 (Peprotech, Rocky Hill, NJ) for various time points, and then fixed with 2% paraformaldehyde in PBS, and permeabilized with 0.1% Triton in PBS for 30 min. Cells were washed in 0.5% BSA in PBS (PBB), blocked with 2% BSA in PBS for 30 min, and incubated with primary antibodies mouse anti-Duffy 2C3 Fy6 (BD Biosciences, San Jose, CA, IgG1), rabbit anti-caveolin-1 (Abcam, Cambridge, MA) or mouse anti-clathrin (Abcam, Cambridge, MA, IgG2b) in PBB at RT for 1 hr. Cells were washed 4 x with PBB then were stained with goat anti-mouse IgG1-Alexa 647, goat anti-rabbit IgG Alexa 488 and goat anti-mouse IgG2b-Alexa 564 (all from Invitrogen, Carlsbad, CA) for 1 hr at RT. Omission of primary antibodies served as controls. Coverslips were mounted to slides using gelvatol (23g polyvinyl alcohol) 2000, 50 ml glycerol, 0.1% sodium azide to 100 ml PBS). Cells were imaged on a Fluoview 1000 confocal microscope (Olympus, Center Valley, PA). Images were taken using a 100x objective using an oil-immersion 100x objective (N.A. = 1.40) digitally zoomed 3x during image acquisition.

For co-localization studies examining Duffy with early endosomal antigen (EEA) and lysosomal associated membrane protein (LAMP), DIH cells were grown in 24 well plates containing 12 mm glass coverslips. Cells were stimulated with or without 100 ng/mL human recombinant CXCL1/GRO-α (Peprotech, Rocky Hill, NJ) or 50 ng/ml PDGF-BB (Millipore, Billerica, MA) for various time points, and then fixed with 2% paraformaldehyde in PBS for 1 hr and permeabilized with 0.1% Triton in PBS for 30 min. Cells were washed in 0.5% BSA in PBS (PBB), blocked with 2% BSA in PBS for 30 min, and incubated with primary antibodies mouse anti-Duffy 2C3 Fy6, rabbit anti-EEA (1∶100 Abcam, Cambridge, MA) and rabbit anti-LAMP (1∶100 Abcam, Cambridge, MA) for 1 hr at RT. Cells were washed 4 x with PBB then were stained with goat anti-mouse Cy3 (1∶1000, Jackson ImmunoReseach), goat anti-rabbit Alexa 488 (1∶500, Invitrogen) and phalloidin-Alexa 647 (1∶250 Invitrogen) for 1 hr at RT. Cells were processed and imaged for confocal microscopy as described above.

### Transmission electron microscopy

#### Cryo-sections

DIH cells were fixed in cryofix (2% paraformaldehyde, 0.01% glutaraldehyde in 0.1 M PBS) and stored at 4°C for 1 hour. Methods for processing ultrathin cryosections have been previously described [56]. Prepared ultrathin sections (70–100 nm) were washed three times with PBS, then three times with PBS containing 0.5% bovine serum albumin and 0.15% glycine (PBG buffer) followed by a 30 min blocking incubation with 5% normal goat serum in PBG. Sections were labeled with rabbit anti-caveolin 1 and mouse anti-Duffy 2C3 Fy6 in PBG for 1 hr. Sections were subsequently washed four times in PBG and labeled with goat anti-rabbit (10 nm) or goat anti-mouse (5 nm) gold conjugated secondary antibodies (GE Healthcare Biosciences, Piscataway, NJ), each at a dilution of 1∶25 for 1 hr. Sections were serially washed in PBG, then PBS, and fixed in 2.5% glutaraldehyde in PBS for 5 min, followed by PBS washes and finally in ddH_2_O. Sections were post-stained in 2% neutral uranyl acetate, for 7 min, washed three times in ddH_2_O, stained 2 min in 4% uranyl acetate, then embedded in 1.25% methyl cellulose. Labeling was observed on a JEOL JEM 1210 electron microscope (Peabody, MA) at 80 kV.

### Pre-Embed ImmunoTEM

DIH monolayers were fixed with 2% paraformaldehyde in PBS for 1 hr. Cells were washed 3 times in PBS, then 3 times within PBS supplemented with 0.5% BSA and 0.15% glycine (PBG) 3×5 min each. Cells were blocked in 20% normal goat serum in PBG for 40 min then washed once with PBG. Primary antibodies (mouse anti-Duffy 2C3 Fy6 in PBG for 1 hr) were added to cells in PBG at a 1∶100 dilution and incubated at room temperature for 4 hr. Cells were washed 4 times in PBG then secondary antibody (goat anti-mouse 5 nm gold conjugate 1∶25 in PBG (Jackson ImmunoResearch Laboratories, West Grove, PA) was added for 3 hr at room temperature. Cells were washed 3 times in PBG, 3 times in PBS, then fixed 1 hr in 2.5% glutaraldehyde in PBS. Cells were then processed for transmission electron microscopy. Monolayers were washed in PBS three times then post-fixed in aqueous 1% osmium tetroxide, 1% potassium ferricyanide for 1 hr. Cells were washed 3 times in PBS then dehydrated through a 30–100% ethanol series then several changes of Polybed 812 embedding resin (Polysciences, Warrington, PA). Cultures were embedded in by inverting Polybed 812-filled BEEM capsules on top of the cells. Blocks were cured overnight at 37°C, then cured for two days at 65°C. Monolayers were pulled off the tissue culture plates and ultrathin cross sections (70 nm) of the cells were obtained on a Riechart Ultracut E microtome, post-stained in 4% uranyl acetate for 10 min and 1% lead citrate for 7 min. Sections were viewed on a JEOL JEM 1011 transmission electron microscope (JEOL, Peobody MA) at 80 KV. Images were taken using a side-mount AMT 2k digital camera (Advanced Microscopy Techniques, Danvers, MA).

### Flow cytometry

DIH cells were pretreated with or without PDGF (Millipore, Billerica, MA) (50 ng/ml) for 10 minutes, then the cells were washed and resuspended in PBS containg 2% FBS. Then the cells were stained with primary antibodies mouse anti-Duffy 2C3 Fy6 (BD Biosciences, San Jose, CA) or Isotype control IgG1 antibody (R&D systems, Minneapolis, MN) for 30 minutes at 4°C. After incubation with secondary antibody anti-mouse IgG-PE for 30 minutes, Flow cytometric analysis was performed on a BD Biosciences FACSCalibur.

In experiments pertaining to surface CXCR2 expression, we obtained whole blood from a healthy volunteer following written informed consent. The Institutional Review Board of University of Pittsburgh approved the studies. The cell pellet was obtained following centrifugation of whole blood at 500 g for 5 minutes. Red blood cells (RBC) were lysed using a commercially available RBC lysis buffer (eBiosciences, San Diego, CA). The cell pellet was washed with PBS x 3, and labeled with either anti-human CXCR2 (BD Biosciences, San Jose, CA) or mouse IgG1 isotype control antibody (BD Biosciences, San Jose, CA) for 30 minutes DIH cells were stained with mouse anti-Duffy 2C3 Fy6, anti human CXCR2 (BD Biosciences, San Jose, CA) or mouse IgG1 isotype control antibody (BD Biosciences, San Jose, CA) for 30 minutes. Samples were analyzed by flow cytometry as stated above.

### SiRNA transfection

DIH cells were transfected with siRNA (80 nM) directed against Caveolin-1(CAV), Clathrin heavy chain (CLAC), Dynamin II (DYN II), Flotillin-1 (FLOT), C-terminal-binding protein-1 (CtBP1) and P21-activated kinase 1 (PAK1), using HiperFect (Qiagen, Valencia, CA) according to the manufacturer's instructions. The transfection efficiency was determined by the protein expression by immunoblotting with the corresponding antibodies 72 hours later, and internalizations assays were performed using nonsilencing SiRNA as controls. All SiRNAs were purchased from Dharmacon and all these SiRNAs are pools of four SiRNA duplexes (smartpools).

### Western blot analysis

Rabbit anti-caveolin-1 (Abcam, Cambridge, MA), mouse anti-clathrin (BD Transduction Laboratories, San Jose, CA), mouse anti-dynamin II (BD Transduction Laboratories, San Jose, CA), mouse anti-flotillin-1 (BD Transduction Laboratories, San Jose, CA), mouse anti-CtBP1 (BD Transduction Laboratories, San Jose, CA), rabbit anti-PAK1 (Cell signaling Technology Inc, Boston, MA) were used for immunoblotting. Proteins were resolved by SDS-PAGE under reducing conditions, transferred to a nitrocellulose membrane, and blocked with 5% nonfat dried milk in TBS containing 0.05% Tween 20 for 1 h. Immunoblotting was performed by incubating membranes in blocking solution containing primary antibodies at 4°C overnight. Following extensive washing with TBS containing 0.05% Tween 20, the membranes were incubated with horseradish peroxidase-conjugated secondary antibodies (Cell Signaling Technology Inc, Boston, MA) for 1 hour at room temperature. An enhanced chemiluminescence detection kit (Thermo Scientific, Rockford, IL) was used to visualize bound antibody.

### Receptor Ligand Internalization

Methods for radioligand internalization were modified from Peiper et al. [15]. All chemical inhibitors were purchased from Sigma (St Louis, MO) unless otherwise indicated. 2-hydroxypropyl-β-Cyclodextrin (10 mM), dynasore (50 µM), amiloride (3 mM), cytochalasin D (2.5 µM), Gö6976 (1 µM; Calbiochem, Gibbstown, NJ) and PDGF-BB (Millipore, Billerica, MA) (50 ng/ml) were used for endocytosis studies. Binding buffer consisted of RPMI 1640 containing 0.2% BSA, and 12.5 mM HEPES buffer, pH 7.4. Acid wash buffer consisted of binding buffer at pH 3.0. DIH or MIH cells (0.2×10^6^ cells) were incubated with 0.2 nM ^125^I-CXCL1/GRO-α (Perkin Elmer, Waltham, MA) or 0.075 µCi ^125^I-Transferrin (Perkin Elmer, Waltham, MA) at 37°C or 4°C. Non labeled excess CXCL1/GRO-α (R&D SYSTEMS, Minneapolis, MN) (1 µM) was added in some experiments. At given time points, the reaction was terminated by centrifugation to separate the cell pellet from supernatant. The pellet was washed twice with either binding buffer or acid wash buffer, followed by a final centrifugation step. The acid-wash step displaced surface bound ^125^I-CXCL1/GRO-α. Radioactivity was measured in counts per minute (cpm) using a gamma counter. The % ^125^I-CXCL1/GRO-α internalized was determined by measuring the average activity of acid-washed wells at 37°C as a percentage of the average activity of non-acid washed wells at 37°C, as previously utilized by others [46], [57]. Average activity of wells incubated at 37°C but acid washed (washed with binding buffer, pH 3) reflected internalized ligand. Average activity of wells incubated at 37°C but not acid washed (washed with binding buffer, pH 7.4) reflected total binding. In order to determine the efficiency of this method, wells reflecting total surface binding (incubated at 4°C, not acid washed) were washed with excess unlabeled ligand (1 µM) and compared to the findings of wells incubated at 4°C and acid washed. Initial results showed that wells incubated at 4°C and acid washed very efficiently removed surface bound ligand. Cholesterol repletion studies have been previously described [58]. Cells were pretreated with 2-hydroxypropyl-β-Cyclodextrin (10 mM) for 30 min followed by incubation in the presence or absence of water soluble cholesterol (400 µg/ml, Sigma, St. Louis, MO). ^125^I-CXCL1 internalization was examined in cells at designated time points as described above.

### Statistics

Student's paired t-test was conducted to compare treatment versus control groups at each time point. For cyclodextrin and cholesterol repletion studies, a two-way analysis of variance (ANOVA) was conducted to compare multiple treatment groups and control across various time points. Statistical analysis was performed using GraphPad Prism5 (GraphPad software, La Jolla, CA).
